# Formaldehyde surrogates in multicomponent reactions

**DOI:** 10.3762/bjoc.21.45

**Published:** 2025-03-13

**Authors:** Cecilia I Attorresi, Javier A Ramírez, Bernhard Westermann

**Affiliations:** 1 CONICET - Universidad de Buenos Aires, Unidad de Microanálisis y Métodos Físicos en Química Orgánica (UMYMFOR), Ciudad Universitaria, Intendente Güiraldes 2160, Pabellón 2, 3° Piso, Ciudad Autónoma de Buenos Aires, C1428EGA, Argentina; 2 Departamento de Química Orgánica, Facultad de Ciencias Exactas y Naturales, Universidad de Buenos Aires, Ciudad Universitaria, Ciudad Autónoma de Buenos Aires, C1428EGA, Argentina; 3 Department of Bioorganic Chemistry, Leibniz Institute of Plant Biochemistry, Weinberg 3, D-06120 Halle (Saale), Germany

**Keywords:** cascade reactions, formaldehyde surrogates, green chemistry, heterocycles, multicomponent reactions

## Abstract

Formaldehyde emerges as a cornerstone in multicomponent reactions, mainly prized for its robust reactivity. Yet, alongside these beneficial traits, this highly reactive C1-building block raises concerns, primarily regarding its toxicity. One notable issue is the challenge of controlling the formation of undesired byproducts during its reactions. This review explores alternative C1-building blocks that serve as surrogates for formaldehyde, aiming to mitigate some of the challenges associated with its use in multicomponent reactions. By identifying these alternatives, toxicity concerns and improved reaction control can be addressed, paving the way for more efficient and sustainable synthetic methodologies.

## Introduction

Organic chemistry is a mature discipline that has undergone significant changes since the term was first coined. Since its inception, organic chemistry has straddled the boundaries between art, creativity, and industrial applications. It is a science that is needed to solve complex retrosynthetic problems and develop molecules in gram to kilogram scales for commercialization. Although numerous challenges still need to be addressed, many are not trivial, and solutions thereof lie beyond known approaches. One of them is still the search for the ideal reaction.

**Scheme 1:**
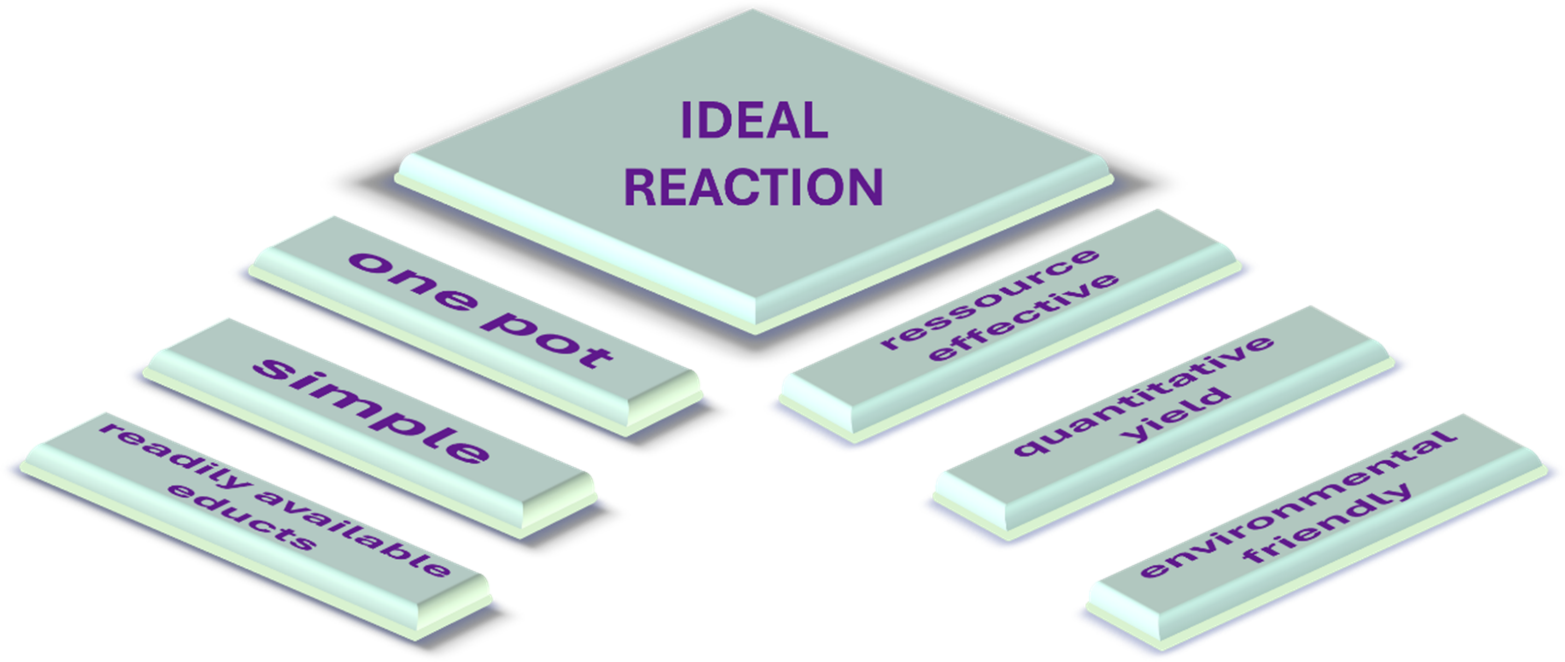
Features of the ideal reaction (redrawn from P. A. Wender et al. [1]).

Scheme 1 exemplifies the ideal reaction, featuring simple reaction conditions (e.g., ambient temperatures, no specifically prepared solvents), one-pot, batch processes leading to tandem or cascade reactions, resource efficiency through the application of the 12 principles of Green Chemistry, and readily available starting materials such as waste materials from food production processes [1–3].

In recent years, multicomponent reactions (MCRs) have become one of the most important synthetic tools coming close to fulfilling the requirements of ideal reactions. They are ideally suited also for the generation of high chemical diversity compounds from simple reagents, either through scaffold diversification or scaffold decoration [4]. In these reactions, three or more compounds react together in one single reaction step to generate a more complex product where most of the atoms of starting materials are present [5]. This high atom economy positions MCRs as ecofriendly (green) reactions because their implementation often implies fewer purification steps to achieve the target molecules, leading to a reduction of waste, when compared to traditional “step by step” synthesis. Another advantage of MCRs is that by selecting appropriate starting materials, follow-up functionalization by, e.g., post-cyclization of the MCR product is possible thus increasing the versatility of these reactions in terms of structural diversity and molecular complexity. In this context, a wide variety of heterocycles and macrocycles as important biological scaffolds have been synthesized [6].

**Scheme 2:**
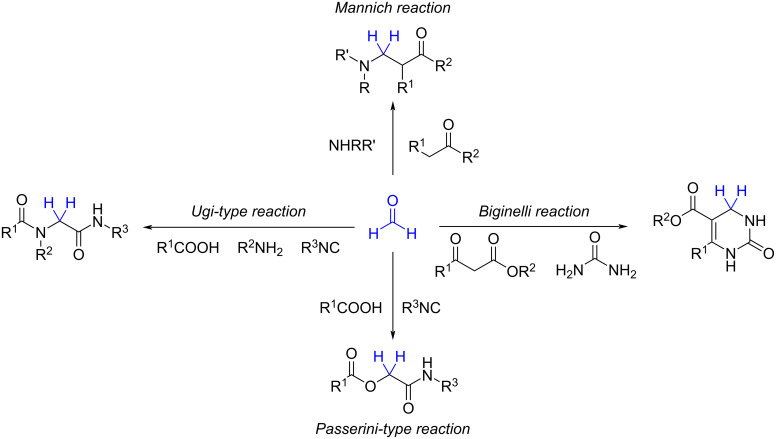
Some of the most popular MCRs with formaldehyde as the carbonyl component.

One of the most important components used in MCRs is formaldehyde, which acts as a highly reactive electrophilic C1 building block in central MCRs reactions such as the Mannich, Biginelli, Ugi, and Passerini reactions (Scheme 2) [7]. In these reactions, formaldehyde undergoes formally the consecutive attack of two other reactants on the carbon atom, due to the readiness for losing a water molecule.

Formaldehyde is commonly used in its polymeric form (paraformaldehyde) or in a 37% aqueous solution (formalin). The exceptional solubility of formaldehyde in water or biobased solvents, such as ethanol and glycerol, enhances the feasibility of green MCRs due to its high reactivity under these conditions [7]. However, this reactivity also increases the potential for the formation of byproducts. Furthermore, formaldehyde is widely recognized to be toxic and is considered carcinogenic by the World Health Organization (WHO) both in solution and in solid forms [8–9].

Given the limitations mentioned above, there has been a critical impetus in recent years to identify formaldehyde substitutes, such as C1 synthons, in MCR. Various efforts have been made to explore alternative reagents that may act as a source of formaldehyde in the reaction medium or lead to the formation of the same final product as that achieved with formaldehyde, but through different reaction mechanisms. Due to the importance of this research, this review aims to summarize and analyze the significant efforts made in this regard in recent years. Major emphasis will be devoted to dimethyl sulfoxide, dihalomethanes, hexamethylene tetramine, and glyoxylates as C1-building blocks, substituting formaldehyde.

## Review

### Methanol as a source of formaldehyde

There are several reports on the use of alcohols under oxidative conditions as aldehyde surrogates in Ugi and Passerini reactions [10]. Oxidation of the alcohol is done in situ to avoid problems regarding the isolation and instability of the aldehyde produced, although undesirable reactions, such as oxidation of the amines or isocyanides or overoxidation of the alcohol, could also be problematic [11–12]. In this regard, several efforts have been made to improve the chemoselectivity of the oxidation step. Among the most relevant examples, *o*-iodoxybenzoic acid (IBX) has been used in Ugi and Passerini reactions to oxidize the suitable alcohol to the desired aldehyde [13]. Alternatively, catalytic amounts of a ternary system (CuCl_2_, NaNO_2_, TEMPO) using molecular oxygen as a terminal oxidant have also been used [12].

**Scheme 3:**
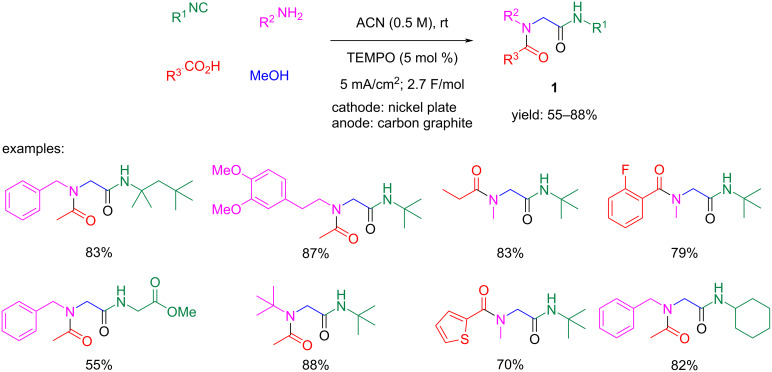
Ugi reaction under a catalyzed electro-oxidation process using TEMPO (2,2,6,6-tetramethyl-1-piperidinyloxyl) as the redox mediator in the oxidation of methanol to formaldehyde.

Nevertheless, neither of these conditions was successful when they were applied to methanol to generate formaldehyde, because overoxidation is an important side reaction in these cases [12–15]. However, in a recent work, Pan et al. could chemoselectively oxidize methanol using a TEMPO-catalyzed electro-oxidation process, even in the presence of oxidizable amines, such as benzylamine, paving the way for the use of methanol as a formaldehyde surrogate in these isocyanide-based MCRs (Scheme 3) and, eventually, in other MCRs where formaldehyde acts as a C1 building block [16]. As can be seen in Scheme 3, under this strategy a wide variety of diamide compounds **1** could be afforded with very good yields applying MeOH as a formaldehyde source in a traditional Ugi reaction.

### Dimethyl sulfoxide (DMSO) as surrogate and source of formaldehyde

Dimethyl sulfoxide is a well-known polar-aprotic solvent with high boiling point that is used in several organic synthetic reactions because of affordable cost and relatively low toxicity. There are numerous examples that show the use of DMSO as a C1 or C2 building block [17–18]. An important drawback of this solvent is the difficulty of its removal from the reaction crude, and extractions with water are commonly employed before purification.

**Scheme 4:**
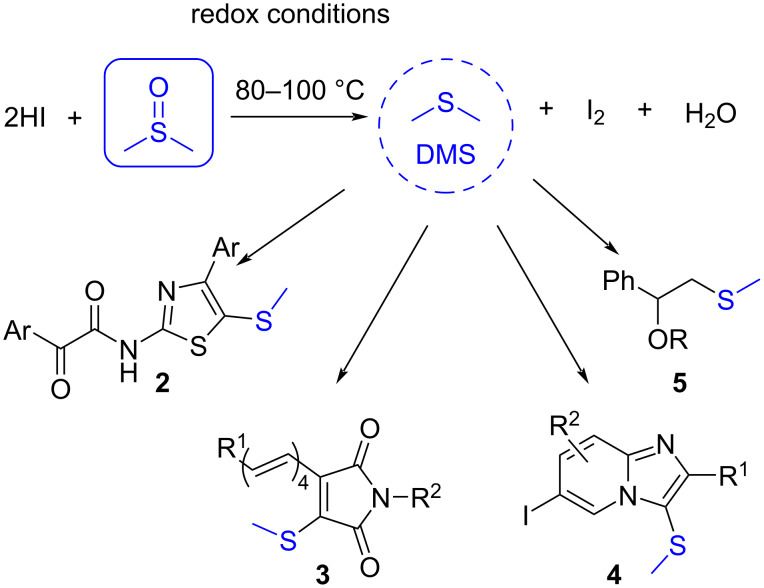
Examples of different products obtained by MCRs in which DMSO serves as -SCH_3_ source.

Depending on the reaction conditions, DMSO can be transformed into different products, e.g., under redox conditions, DMSO can decompose into DMS (dimethyl sulfide), which then can act as a nucleophile in several MCRs (Scheme 4) [19–22]. In this context, DMSO can be used as a source of the methylsulfanyl (-SCH_3_) group after DMS follows a nucleophilic addition or substitution, allowing one to obtain different types of products (products **2** to **5**, Scheme 4).

**Scheme 5:**
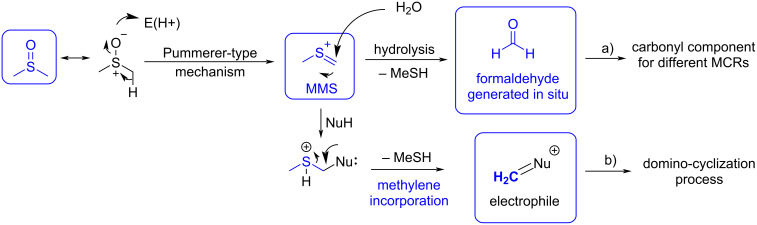
Mechanism of the decomposition of DMSO under acidic or thermal conditions. a) In situ generation of formaldehyde after hydrolysis of MMS. b) Reaction with other nucleophiles (Nu^−^).

In other cases, under acidic and thermal conditions, DMSO can undergo a Pummerer-type process in which, upon activation of the sulfoxide oxygen, a reactive methyl(methylene)sulfonium ion (MMS) is formed (Scheme 5) that acts as an active electrophile. Depending on the nucleophilic species present in the reaction medium, MMS can act as a methylene source. Under hydrolysis conditions, it can be a source of formaldehyde (Scheme 5a), but with other nucleophiles, after nucleophilic addition, the sulfide group can work as a leaving group, allowing for a sequential domino process such as an aza-Prins cyclization [23] and aza-Diels–Alder reaction [24], where MMS serves as direct source of the methylene (-CH_2_-) group (Scheme 5b).

In both cases, the resulting reactive species (MMS or formaldehyde) can participate as electrophilic component in several MCRs as C1 building block.

#### Synthesis of heteroaromatic systems

One of the most powerful applications of MCRs is the synthesis of heterocyclic compounds and several reports in recent years showed the advantages of using this tool as a synthetic strategy to generate complex molecular scaffolds with medicinal relevance [25–27].

**Scheme 6:**
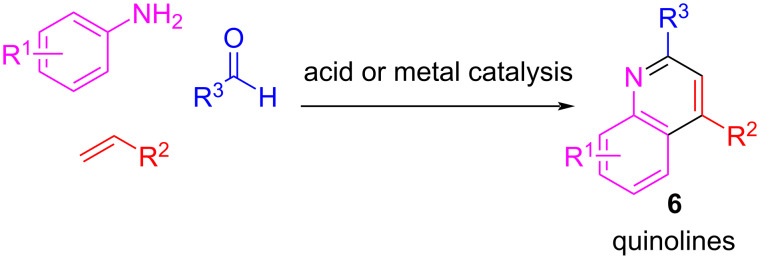
Povarov multicomponent reaction to quinolines.

In this context, quinoline and its derivatives are privileged structures in several natural products and biologically active compounds, rendering this scaffold an important synthetic target. An attractive strategy to afford tetrahydroquinolines and quinolines is the Povarov reaction, a type of aza-Diels–Alder reaction between an imine and an alkene (Scheme 6). Very successfully, the multicomponent version of the Povarov reaction using aldehydes, anilines, and alkenes has been explored in a three-component cascade reaction to quinolines [28–31] (Scheme 6). Furthermore, protocols have been developed in which the alkene compound has been replaced with other surrogates for electrophilic addition, such as ketones [32–34]. In the case of the carbonyl component, it is generally an aromatic aldehyde [30,33–34] and there are not many reports on using formaldehyde in the Povarov reaction.

**Scheme 7:**
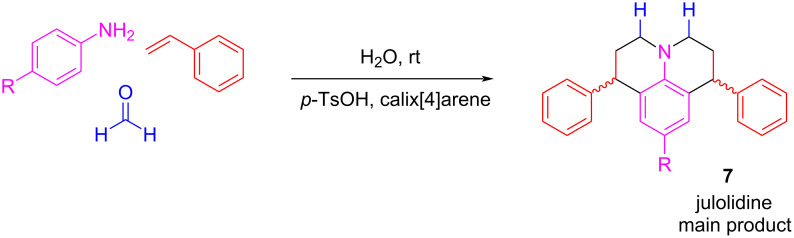
Example of the Povarov reaction with formaldehyde with a julolidine derivative as main product.

In a remarkable example, when formaldehyde was used, the reaction did not provide the desired quinoline **6** as the main product but rather julolidines **7** (Scheme 7) [31].

However, the use of paraformaldehyde and glycine can produce the desired products with low yields, but very expensive catalysts and complex separation processes are required [32,35–36].

**Scheme 8:**
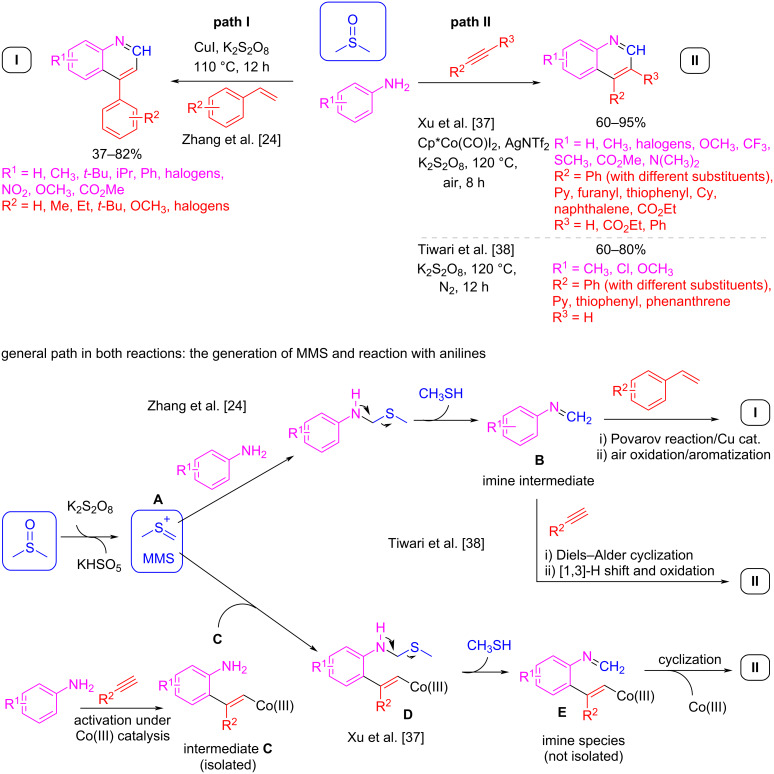
Povarov multicomponent reaction to quinoline derivatives **I** and **II** using DMSO as formaldehyde surrogate.

In this context, DMSO has been used as an alternative to formaldehyde for the MCR synthesis of a wide variety of quinolines and related compounds. For example, Zhang et al. showed that starting from anilines and substituted styrenes while using K_2_S_2_O_8_ for the conversion of DMSO to MMS, a wide range of 4-arylquinolines can be synthesized (Scheme 8, path I) [24]. In this reaction, the persulfate ion generates the thionium ion (MMS), which is trapped by a nucleophilic aniline. The loss of methyl sulfide generates an imine intermediate **B**, which, in turn, reacts with substituted styrene under copper(I) catalysis to give the target compounds via a Povarov reaction. A further aromatization process yields product **I** (Scheme 8, path I). In a closely related approach, the same group reported on the synthesis of quinolines from anilines and alkynes [37]. In this case, the alkyne first reacts with the aniline under cobalt(III) catalysis, and the resulting intermediate **C** then attacks the thionium ion **A**. Quinolines of general structure **II** are formed after the loss of methyl sulfide from intermediate **D**, followed by final cyclization of intermediate **E** (Scheme 8, path II).

Additionally, the Tiwari group developed a metal-free protocol using only K_2_S_2_O_8_ as an oxidant for the activation of DMSO to MMS (Scheme 8, path II) [38]. Under these conditions, an alternative mechanism arises in which the imine intermediate **B**, formed as previously stated through reaction between the aniline compound and MMS, undergoes an aza-Diels–Alder cyclization with the alkyne, and after oxidation and aromatization steps generates quinoline **II**. Unfortunately, under these gentle and greener conditions, aliphatic alkynes remain unreacted, compared to the metal-catalyzed version developed by Xu et al. [37] with which a wide range of aromatic and aliphatic disubstituted alkynes were reactive, resulting in a greater diversity of quinolines **II**.

In both cases, regardless of the catalyst used, the MCR tolerates a wide variety of anilines that have either electron-donating or electron-withdrawing groups with various substitution patterns in the aromatic ring, allowing a high variety of quinolines of general structure **I** and **II**.

**Scheme 9:**
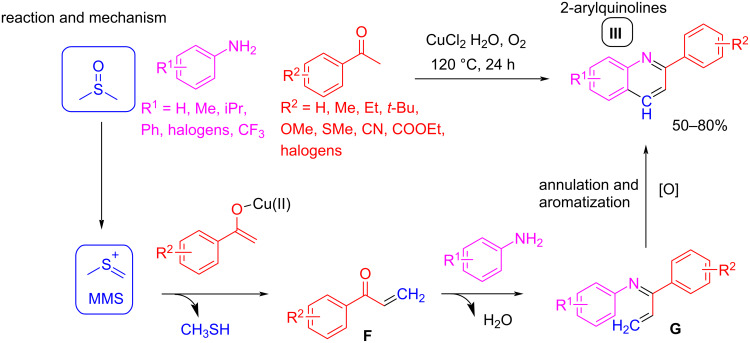
Example of a Povarov three-component reaction with change of catalyst, yielding regioisomer **III**. In this case, the methylene group is assembled at the C4 position.

In a related work, quinolines of structure **I** (Scheme 8) could be obtained by similar reaction pathways. Jadhav et al. proposed a three-component cascade reaction between anilines, methyl aryl ketones, and DMSO under iron(III) catalysis and using K_2_S_2_O_8_ for its activation [39]. The proposed mechanism is very close to those described above, with the methyl aryl ketone taking part of the reaction in place of the styrene component in the Povarov cyclization. In this case, the imine reacts with the enolate of the ketone, which is stabilized by coordination with Fe(III), resulting in the formation of the C–C bond. A further oxidative aromatization process affords compound **I**. Compared to the protocol developed by Zhang et al. [24], the reaction is less regioselective, as Troger’s base derivatives are isolated as side-products. Interestingly, when Liu et al. [40] modified this reaction by using a copper(II) catalyst under aerobic oxidative conditions, the regioisomers **III** (2-arylquinolines) were obtained (Scheme 9).

To rationalize this singular result, the authors proposed a mechanism in which, after MMS formation, this reactive species is subsequently captured by the stabilized Cu(II) enolate of the ketone, to provide an α,β-unsaturated ketone intermediate **F**. This compound condenses with the aniline component to give an imine **G** that follows a cyclization and aromatization cascade reaction, affording 2-arylquinolines **III**.

All of these examples showed the versatility of DMSO as a methylene source in the synthesis of substituted quinoline compounds (structures **I**, **II,** and **III**; Scheme 8 and Scheme 9), using different substrates (such as styrenes, alkynes, and methyl aryl ketones) and consequently, different catalytic strategies to afford the electrophilic addition on the final cyclization step.

**Scheme 10:**
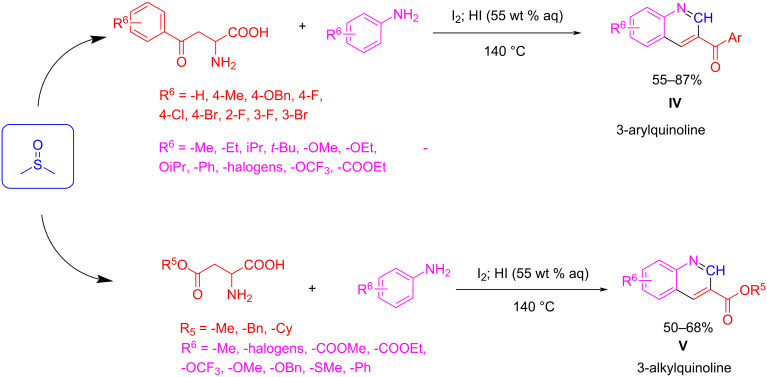
The Povarov three-component reactions carried out under acidic catalysis to afford quinoline regiosiomers **IV** and **V**.

Finally, other examples show the synthesis of 3-aryl and alkyl quinoline-3-carboxylate derivatives under acid catalysis for the activation of DMSO via the Pummerer reaction (Scheme 10). In these cases, phenylalanine and aspartate derivatives react with aniline compounds to provide quinoline regioisomers **IV** and **V**, respectively [41]. These reactions are suited for a broad range of reactants with both electron-donating and electron-withdrawing groups.

**Scheme 11:**
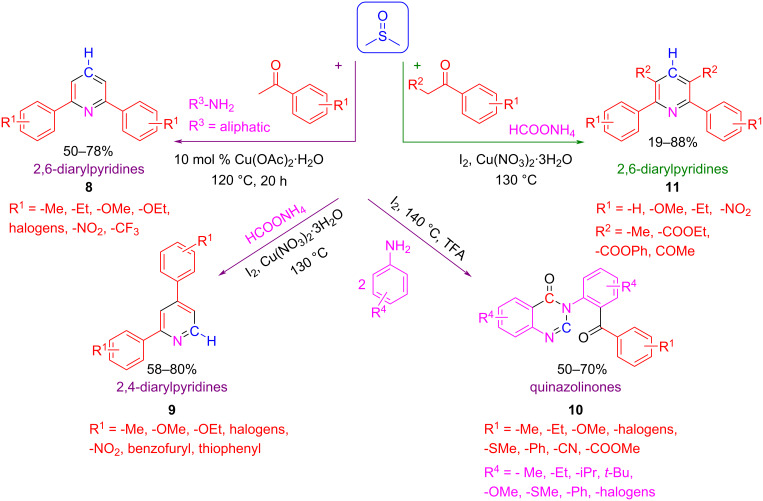
Different MCR routes involving DMSO to synthesize complex heterocycles such as diarylpyridines and quinazolinones.

DMSO activation through a Pummerer reaction (as exemplified in Scheme 10 above) and its use in heterocycle multicomponent synthesis is not limited to quinolines, as the reaction to diarylpyridines [42–43], quinazolinones [44], and pyrazoles [45] is also described by this approach (Scheme 11). These examples deserve further discussion.

Quinazolinones **10** can be synthesized from substituted acetophenones and aromatic amines (Scheme 11) [44]. On the other hand, diarylpyridines **8**, **9**, and **11** can be obtained from acetophenones, but using aliphatic amines or ammonium formate as the nitrogen source [42–43]. In all these cases, the activation of DMSO was carried out via copper catalysis or iodine–acid catalysis. Interestingly, when aliphatic amines are employed (R^3^ = *n*-Pr, *n*-Bu, product **8**) only the N atoms are incorporated in the structure of the final product, probably because the high temperature favors the elimination of the alkyl group. The reaction works very well for a wide variety of functional groups and substitution patterns in the aryl methyl ketone substrate, affording the desired heterocycle with good yields. Even heteroaryl methyl ketone and 1,3-dicarbonyl compounds work very well under these reaction conditions, leading to more complex heterocycle products of general structure **9** and **11**. Interestingly, Wu et al. demonstrated that the reaction did not proceed when paraformaldehyde is used as the C1 synthon, indicating that formaldehyde is not involved in the reaction [43]. Furthermore, in isotope labelling studies using DMSO-*d*_6_ all of these authors confirmed, after analyzing the position of the deuterium atoms in the final compound, that the methylene unit incorporated into the heterocycle came from DMSO.

**Scheme 12:**
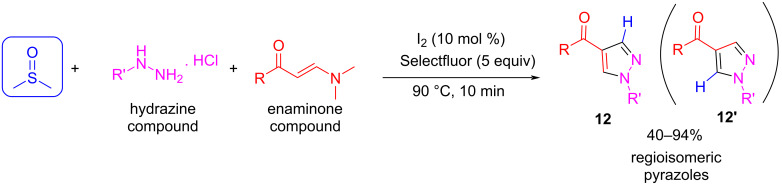
Pyrazole synthesis by a three-component reaction using DMSO as a source of a C-1 unit.

In the case of pyrazole synthesis, Guo et al. proposed a three-component cascade reaction of enaminones, hydrazines, and DMSO (Scheme 12) [45]. In this case, the reaction works well under metal-free conditions using iodine as the catalyst. Remarkably, the activation of DMSO was accomplished using Selectfluor, and in this case, DMSO is the source of a C-1 unit. It is important to note that the reaction could be performed using formaldehyde in only minor yields. This can be explained in terms of the regiospecificity of the reaction: when DMSO is used as the C1 synthon, two different ways of transferring the CH group at different positions in the pyrazole ring (**12** and **12’**) are allowed. However, this cannot be accomplished in the case of formaldehyde, making the reaction more regioselective.

In the cases shown above, we discussed different ways to activate DMSO. In almost all of them, MMS appears as the reactive species due to its high electrophilicity, which allows for an efficient nucleophilic addition, and the presence of an excellent leaving group (methyl sulfide) which permits further transformations during the domino sequences. This dual effect of MMS makes DMSO an interesting C1 synthon that works not only as a source of a methylene unit (-CH_2_-) in terms of formaldehyde surrogate, but also as a promotor of the cyclization process producing various highly diverse nitrogen-containing heterocycles, which are valuable scaffolds in medicinal chemistry [46].

#### Synthesis of non-aromatic heterocycles

**Scheme 13:**
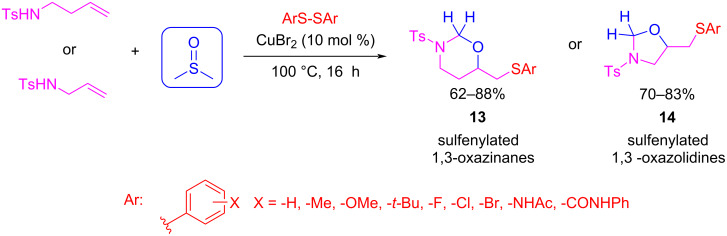
Three-component reactions for the synthesis of aliphatic heterocycles **13** and **14** using DMSO as a formaldehyde surrogate.

As stated above, DMSO can also hydrolytically decompose to formaldehyde. There are many examples of reactions in which DMSO is used as a formaldehyde surrogate, which have been summarized in recent reviews [17–18,47]. We would like to highlight those examples where MCR reactions are involved. In this context, Zhong’s group developed the synthesis of sulfenylated 1,3-oxazinanes **13** and oxazolidines **14** via a thia-aza-Prins cyclization reaction of homoallylic amines with disulfides and DMSO under copper catalysis, where DMSO acts as a solvent and a formaldehyde source at the same time (Scheme 13) [48]. This protocol represents a versatile method for the construction of oxygen-containing heterocycles, in which the oxazinane skeleton is an interesting scaffold for the design of synthetic routes for drug targets.

The reaction works very well with a broad range of aromatic disulfides. *Ortho, meta,* and *para*-substituents with different electronic properties afford moderate to excellent yields. The reaction fails only when aliphatic disulfides are used because of the higher Lewis basicity of the alkylated sulfur atom, which poisons the copper catalyst. When the reaction was carried out with paraformaldehyde and other solvents (such as DMF, 1,4- dioxane, toluene, and DCE) the yield was very low (between 0–34%), but when DMSO is used as solvent and reagent, the yield was greatly improved.

**Scheme 14:**
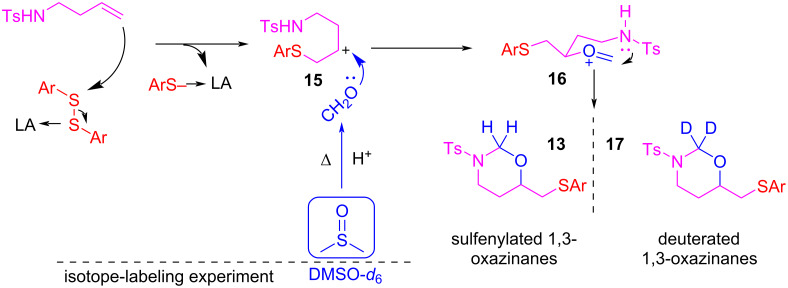
Proposed mechanism for the 3CR between homoallylic amines, disulfides, and DMSO.

The proposed mechanism involves the activation of the disulfide component by CuBr_2_ as the Lewis acid (Scheme 14). The copper(II) center coordinates the sulfur atom of the disulfide allowing for the electrophilic addition to the alkene moiety of the amine. The resulting stabilized carbocation **15** is then captured by formaldehyde (generated in situ from DMSO) leading to an intermediate oxocarbenium **16** that undergoes a cyclization to obtain the sulfenylated oxazinane derivative **13**.

In isotope labelling experiments using DMSO*-d*_6_ the expected deuterated product **17** is obtained, confirming the in situ generation of formaldehyde from DMSO as the source of the methylene group. Interestingly, the reaction gives better yields under these conditions than that observed when paraformaldehyde is used.

#### DMSO in Mannich-type MCRs

**Scheme 15:**
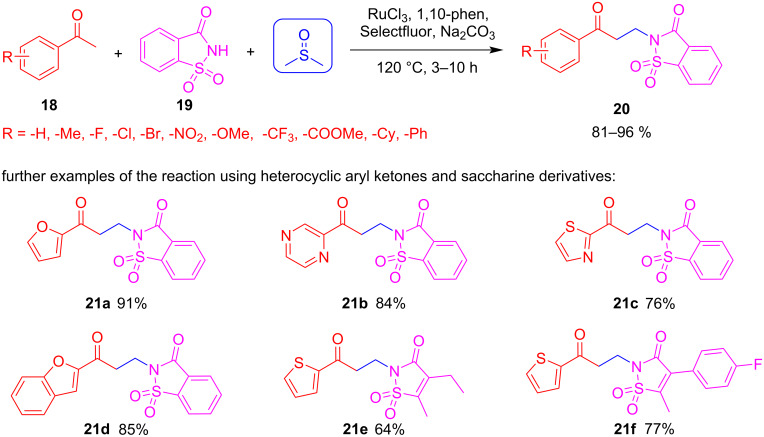
Mannich-type reaction using DMSO as formaldehyde surrogate.

Sun et al. developed a three-component Mannich-type reaction under oxidative and catalytic conditions that allows the coupling of aryl ketones **18** and saccharine (**19**) using DMSO as the solvent and the source for a methylene bridge linking the two building blocks (Scheme 15) [49]. Following this strategy, they synthesized a large library of compounds of general structure **20**. Furthermore, they extended the reaction to heteroaryl ketones to obtain a heterocycle containing β-amino ketones **21a**–**f** [50]. The reaction tolerates a wide range of functional groups at the substrates, giving a wide structural scope to the resulting compounds.

**Scheme 16:**
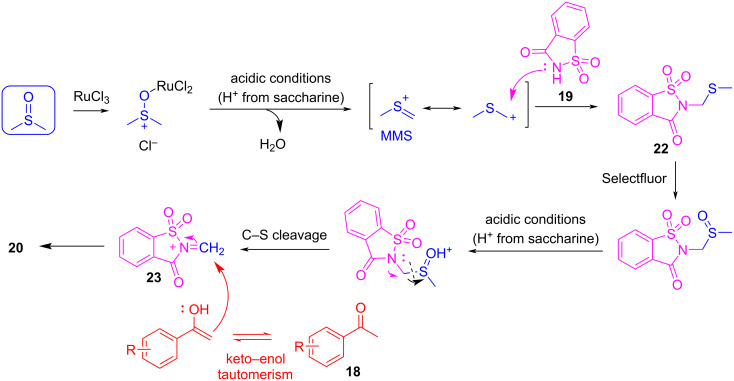
Mechanism for the 3CR-Mannich-type reaction between aryl ketone **18**, saccharine (**19**), and DMSO. The generation of C-1 unit MMS arises from the decomposition of DMSO under acidic and catalytic conditions.

Once again, DMSO was confirmed as the origin of the methylene bridge by isotope labelling experiments using DMSO-*d*_6_. The proposed mechanism comprises the activation of DMSO by a Ru(III) catalyst and the role of Selectfluor working as the oxidant that allows the "activation" of the methyl sulfur group in intermediate **22** for the cleavage of the C–S bond. In the end, the C–C bond is formed between intermediate **23** and the enol form from the methyl ketone **18**. Sodium carbonate is added to prevent too much acidification of the reaction medium and to deprotonate the NH that traps the sulfonium ylide (Scheme 16).

**Scheme 17:**
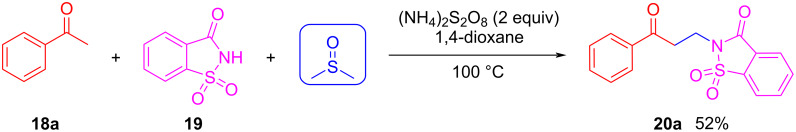
Mannich-type reaction using DMSO as formaldehyde surrogate and under oxidative activation.

In an independent work, Mhaske et al. proposed an alternative methodology to furnish β-amino ketone **20a** (Scheme 17), using DMSO as a formaldehyde surrogate but with activation via ammonium persulfate (APS), avoiding the use of transition-metal catalysis [51]. In this case, the mechanism appears to proceed through the formation of free radical species, where APS plays the role of oxidant and radical activator of DMSO, generating reactive radical species of DMSO or dimethyl sulfone that react with the nitrogen of saccharine compound **19** without the need for a catalyst.

**Scheme 18:**
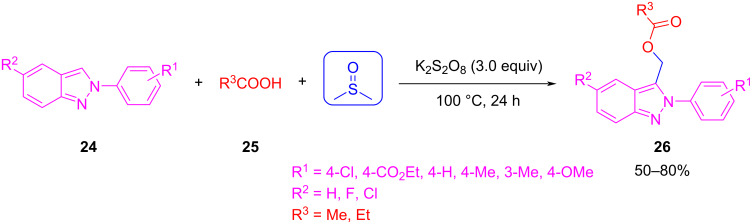
Three-component reaction between an indazole, a carboxylic acid, and DMSO.

Another recent example for the use of DMSO as C1 synthon was reported by Bhattacharjee et al. They used DMSO in a 3CR to install a methylene unit between an indazole **24** and a carboxylic acid **25** (Scheme 18) [52]. Under radical conditions using K_2_S_2_O_8_, they obtained a series of carboxylic acid esters of indazoles **26**.

In all these multicomponent reactions DMSO was applied to install a C1-bridge between two structural units that already have a heterocycle moiety incorporated.

### Dihalomethanes

Dihalomethanes are good solvents for several organic syntheses. Their low boiling points and polar non-protic nature make them suitable dissolving agents for a wide range of reagents. Furthermore, the polar bond C–X can be activated by different chemical procedures (metal or acid catalysis, for example) and serves as a reactive center. In this regard, dihalomethanes can act as halogen donors in nucleophilic reactions or as methylene sources in electrophilic reactions [53–57]. In the latter case, they are employed as C1 building blocks, with abundant examples in the literature [55–57]. This section will focus on the most important uses of dihalomethanes as formaldehyde alternatives in MCR reactions applied to the synthesis of propargylamines and aminophosphonates. We will discuss the reaction conditions, mechanisms, and scope.

#### Synthesis of propargylamines

**Scheme 19:**
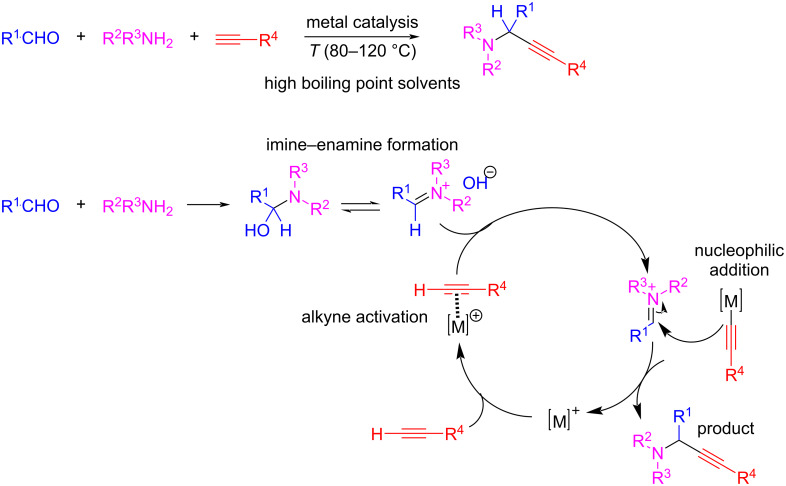
Amine–aldehyde–alkyne (AAA) coupling reaction and plausible mechanism.

Propargylamines are essential building blocks for the synthesis of natural products or biologically active compounds in medicinal chemistry [58]. One of the most effective strategies for their synthesis is the addition of alkynes to imines or enamines, which is typically carried out under metal catalysis and elevated temperatures. This process requires the use of high boiling point solvents such as toluene, dimethylformamide (DMF), dimethyl sulfoxide (DMSO), or dioxane to achieve high yields. The assumed mechanism is initiated by activation of the C–H bond of the terminal alkyne by a metal catalyst. The resulting metal acetylide reacts with the imine/enamine through a nucleophilic addition. Because imines/enamines are available from the reaction between aldehydes and amines (primary or secondary), the reaction is called AAA coupling: amine–aldehyde–alkyne coupling (Scheme 19).

In this context, the use of formaldehyde as a C1 building block is suitable despite the unstable aminol intermediate. There are several examples showing how formaldehyde or paraformaldehyde can be used in the AAA coupling of three components for the synthesis of propargylamines [58–61]. However, the problems associated with the stability of the imine/enamine intermediate generated from formaldehyde must still be addressed.

**Scheme 20:**
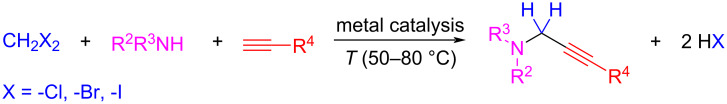
AHA coupling for the synthesis of propargylamines using dihalomethanes as C1 building blocks.

Several attempts have been made to replace formaldehyde with dihalomethanes. These can react with certain secondary amines to generate stable aminals, which increase the rate of reaction at higher temperatures or pressures. This gives rise to products derived from iminium ions and therefore, dihalomethanes can function as aldehyde equivalent in reactions where iminium species are involved. In this case, the C–X bond of the dihaloalkanes can be activated by metal catalysis, allowing the incorporation of the C1 building block by a mechanism that does not involve the preformation of an imine/enamine intermediate. These are the basis for the AHA coupling: amine–haloalkane–alkyne coupling for the synthesis of propargylamines by the activation of both C–H and C–X bonds by metal catalysis (Scheme 20).

In general, the activation of both CH and CX bonds is accomplished by homogeneous metal catalysis with CuCl being the most used one. This is usually done at a load of 5 mol % in CH_3_CN as solvent and CH_2_Cl_2_ as a C1 synthon, under moderate reaction conditions (60 °C). Consequently, this synthetic strategy is less dependent on temperature compared to AAA coupling [62].

Several reports also showed that gold (as AuCl_3_, 5 mol %) [63], indium (as In_2_O_3_ nanoparticles, 5 mol %) [64], iron (as FeCl_3_, 20 mol %) [65], cobalt (as CoBr_2_, 10 mol %) [66], and nickel (as Ni(py)_4_Cl_2_, 15 mol %) [67] can act as metal catalyst for the 3CC reaction. In all these cases, the temperature was lower (usually between 60–80 °C) compared to the AAA coupling, except for iron, where the temperature must be increased to 100 °C. This can be explained in terms of the activation of both the C–H and C–X bonds by metal catalysis, which is not the case in AAA couplings, where only the C–H bond is activated, making the last step (nucleophilic addition) more temperature dependent. However, in the case of nickel catalysis, during AHA coupling, a suitable ligand, such as bipyridine, is needed for the in situ formation of a metal complex that activates the C–H and C–X bond [67].

**Scheme 21:**
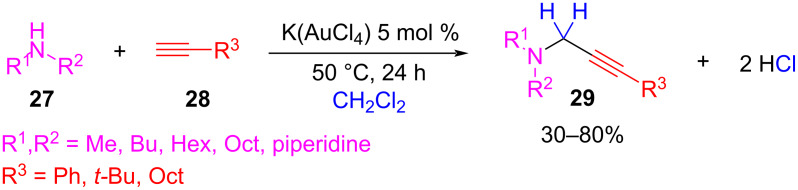
AHA coupling using CH_2_Cl_2_ as both solvent and methylene source.

The solvents used most in the AHA coupling are CH_3_CN [62,65–67], DMSO [64], water, and even neat conditions [62]. In some cases, the same dihalomethane can be used as both the reactant and solvent. For example, Aguilar et al., proved that when using CH_2_Cl_2_ as both a solvent and a C1 source, they could obtain propargylamines **29** with good yields from secondary amines **27** and alkynes **28** (Scheme 21) [63]. In this case, the catalysis was accomplished by a gold(III) catalyst.

In general, CH_2_Cl_2_ is preferentially chosen as the dihalomethane compound due to economic reasons and ease of access, but some reports also used CH_2_Br_2_ and CH_2_I_2_ with good results [62,66–67].

In some cases, the addition of a non-nucleophilic base is needed to neutralize the HCl generated during the reaction (Scheme 20). For example, Yu et al. demonstrated that the reaction yield could be significantly increased when the reaction was performed in the presence of one equivalent of DBU [62]. They argued that organic bases such as DBU are superior than inorganic bases in improving the yields, probably because of the low solubility of the last ones in the reaction system. In the same way, other bases such as DABCO [64], DBU [66], and TMG (1,1,3,3-tetramethylguanidine) [65,67] were shown to be useful for metal activation of the C–H alkyne deprotonation.

**Scheme 22:**
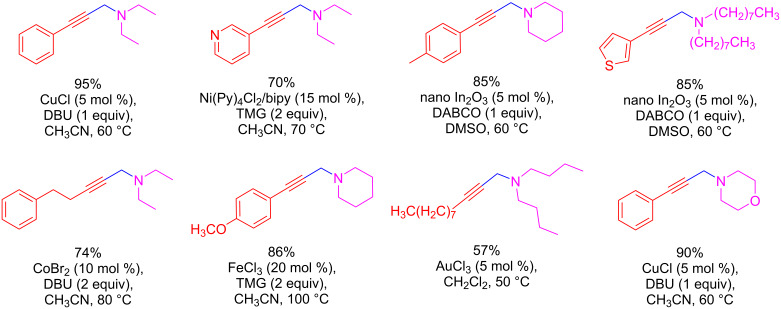
Examples of propargylamines synthesized under catalytic AHA protocols.

The use of dihalomethanes in these reactions provides a wide scope in terms of the type of alkyne and amine that can be employed (Scheme 22) [62–68]. In general, aromatic and aliphatic alkynes, even with electron-donating or electron-withdrawing groups, work well under these conditions. However, the amine component does not react when primary or aromatic secondary amines are used. It works very well with cyclic or acyclic aliphatic secondary amines, such as piperidine or dibutylamine. In all cases, the yields are between 60–95% for all metal catalysis conditions.

**Scheme 23:**
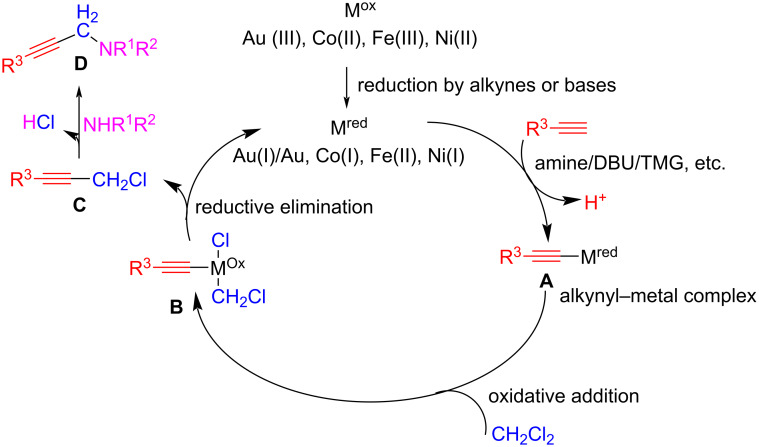
Proposed mechanism for the synthesis of propargylamines using dichloromethane as a C1 source.

The most widely accepted mechanism is as follows: the alkynyl C–H bond is activated by the metal catalyst (Scheme 23). The metal can be added in its proper oxidation state (such as Cu(I)) or generated in situ (as in the case of Au(I), Co(I), Fe(II) and Ni(I)) by reducing the suitable salts (containing Au(III), Co(II), Fe(III), and Ni(II), respectively) at the beginning of the catalytic cycle. The metal in its reduced species (depicted in Scheme 23) activates the C–H bond of the alkyne. After this activation step, a weak base (like DBU, TMG, or even the same amine component) deprotonates the terminal alkyne, generating the metal acetylide derivative **A**, which is the active nucleophilic species in the reaction. Intermediate **A** undergoes an oxidative addition by the dihaloalkane, generating intermediate **B**. This undergoes reductive elimination to afford propargyl halide **C**. Finally, intermediate **C** reacts with the secondary amine to give the propargylamine product **D**.

As outlined in the catalytic cycle, the presence of a base plays a dual role: co-activation of the alkynyl C–H bond through deprotonation and trapping of the HCl produced during the last step (nucleophilic substitution in intermediate **C** by the amine compound). Depending on the conditions, the role of the base can be fulfilled by the amine itself [63] or by the addition of another base such as DBU [66], DABCO [64], or TMG [65].

This mechanism is supported by experimental evidence for the formation of intermediates. Gao et al. studied the generation of metal acetylide **A** by IR spectroscopy [65]. When subsequent additions of 1 equiv FeCl_3_ were made in a solution of alkyne and TMG, the C–H stretch peak at 3277 cm^−1^ started to decrease as the temperature increased from 30 to 100 °C. This result suggests the generation of the Fe acetylide intermediate type **A**. On the other hand, Tang et al. prepared a derivative of intermediate **C** and subjected it to reaction with piperidine under optimized reaction conditions (DBU in MeOH at 80 °C) and obtained the propargylamine product **D** [66]. Based on this result, the implication of intermediate **C** in the reaction mechanism was demonstrated.

**Scheme 24:**
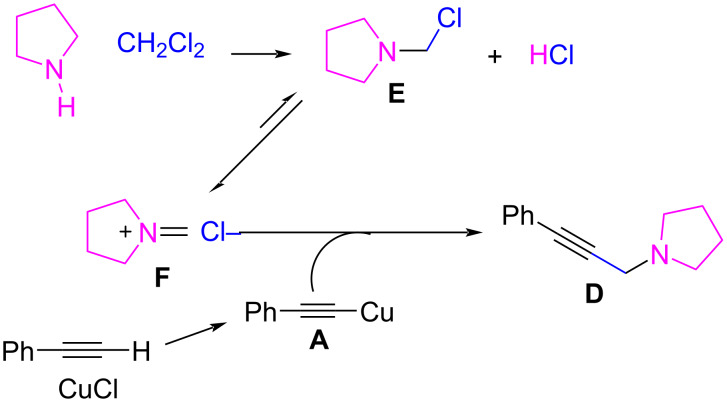
Mechanism proposed for the generation of the aminal intermediate **E** by Buckley et al. [68].

However, an alternative pathway from intermediate **A** to the propargylamine product has been proposed when copper is used as the catalyst. In this case, the activation of the C–X bond of the dihaloalkane requires not only the metal catalyst but also the amine compound for the reaction to proceed [62]. It was suggested that the propargylamine product is formed directly from the activated intermediate **B** by the amine. This can be deduced from experiments performed starting from an alkyne and dichloromethane under these catalytic conditions (CuCl, 5 mol % and 1 equiv of DBU), in which intermediate **C** is not produced if the amine compound is absent, thus suggesting that the amine is necessary for the activation of the C–X bond. Besides, an alternative mechanism has been proposed similar to the AAA coupling, in which the copper catalyst (CuCl, 15 mol %) only activates the C–H bond of the terminal alkyne, and the resulting nucleophile **A** reacts with the iminium ion **F** generated from CH_2_Cl_2_ and the secondary amine via an aminal intermediate **E** (Scheme 24) [68].

However, Yu et al. found no evidence for the formation of an aminal intermediate **E** or the enamine derivative when they tried to obtain it from the reaction of CH_2_Cl_2_ and diethylamine in the absence of alkyne under suitable conditions (CuCl, 5 mol %, DBU 1 equiv, CH_3_CN, 60 °C) [62]. Only in some cases can the reaction of CH_2_Cl_2_ and certain cyclic secondary amines such as piperidine or pyrrolidine afford an aminal or an iminium ion, in general under high temperature or pressure conditions, which is not the case for the AHA coupling [69–70]. Despite these cases, the absence of evidence in terms of iminium ion generation confirms that the AHA coupling reaction by metal catalysis is an option to avoid the problems regarding iminium or aminal stability. This is an important issue in AAA coupling when formaldehyde is used as the C1 source.

Finally, a later work proposes a catalysis-free protocol for the synthesis of propargylamines by an AHA coupling reaction [71]. Here, the reaction is carried out under mild conditions (CH_2_Cl_2_, 70 °C) without the need for catalysis or additional base for the activation of C–H and C–X bonds of the alkyne and dihalomethane compounds, respectively. However, this strategy has a limited scope in terms of the alkyne compound as no reaction is observed with aliphatic alkynes. Furthermore, the yields are between 50–80%, which are slightly lower than those under metal-catalyzed conditions. Nevertheless, these results open the door to further studies aimed at developing more efficient, non-catalyzed synthetic procedures for obtaining propargylamines.

#### Synthesis of α-aminophosphonates

**Scheme 25:**
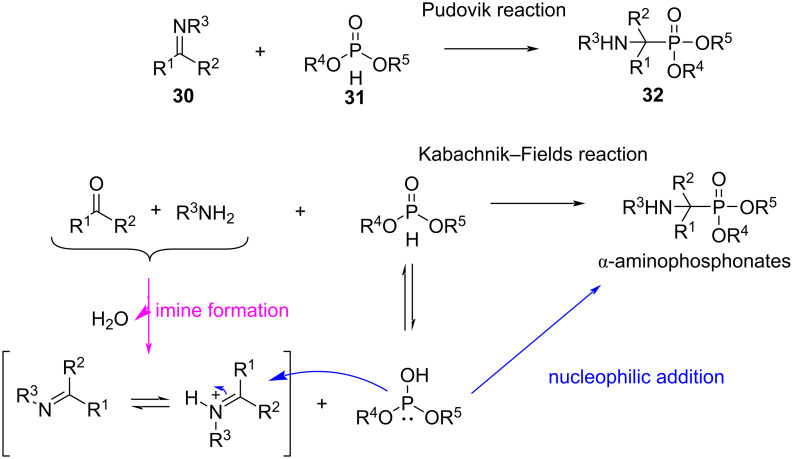
Pudovic and Kabachnik–Fields reactions for the synthesis of α-aminophosphonates.

One of the most robust methods for the synthesis of α-aminophosphonates is the Pudovik reaction, along with its multicomponent version, the Kabachnik–Fields reaction (Scheme 25) [72–73].

**Scheme 26:**
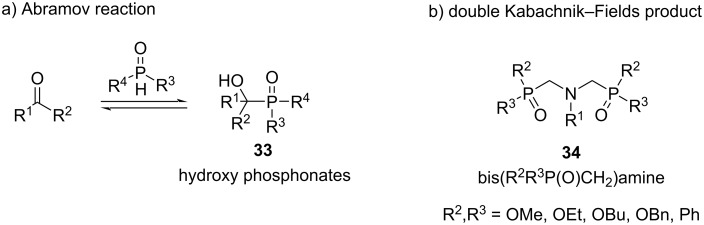
a) Abramov side reaction that generates α-hydroxy phosphonate as a byproduct during the Kabachnik-Fields reaction. b) Bis(phosphorylmethyl)amine derivative product obtained from double Kabachnik–Fields reaction using formaldehyde.

In the Pudovik reaction, a dialkyl phosphite **31** containing a PH bond adds to the C=N bond of a preformed imine **30**, while in the three-component Kabachnik–Fields reaction, the imine, which is generated in situ from a carbonyl compound and an amine, reacts with the dialkyl phosphite to produce the desired α-aminophosphonates **32** (Scheme 25) [72,74]. This reaction has been reported to proceed with a wide variety of aliphatic aldehydes, in particular formaldehyde, under different reaction conditions with or without solvent or catalysis [72,74–76]. However, a significant problem with the Kabachnik–Fields reaction arises from the fact that dialkyl phosphites can also undergo an addition to the C=O bond of the carbonyl component (Abramov reaction) giving α-hydroxy phosphonates **33** as byproducts (Scheme 26a) [72]. Competition between the two nucleophiles for the electrophilic carbonyl compound depends on their relative reactivity [74,77] and this lack of chemoselectivity becomes important when formaldehyde is used. Moreover, primary amines (alkyl- and arylamines) can also react with two equivalents of both the formaldehyde and the P(O)H compound. In this case, a double Kabachnik–Fields condensation gives bis(phosphorylmethyl)amines **34** as possible byproducts (Scheme 26b) [76,78].

**Scheme 27:**
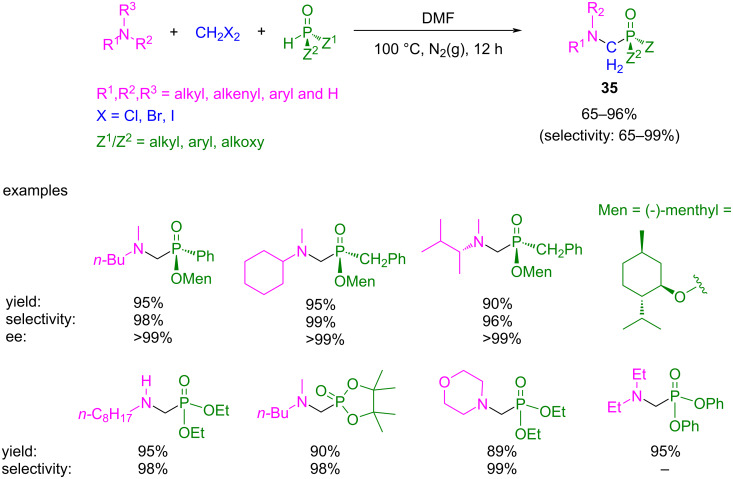
Catalyst-free three component reaction to afford α-amino phosphorus product **35** using 1,1-dihaloalkanes as a C1 building source.

Because of the tendency of formaldehyde to generate these byproducts, several efforts have been made to optimize the chemoselectivity of the Kabachnik–Fields reaction. In this context, Zhao et al. proposed a more selective strategy for the synthesis of α-amino phosphorus compounds using dihalomethanes (Scheme 27) [79]. They developed a three-component reaction between amines (mainly tertiary amines), a dihalomethane, and a P(OH) species (like phosphonate, phosphinate, or secondary phosphine oxide) under catalyst-free conditions to afford α-amino phosphorus compounds **35**. The products are very appealing biologically relevant scaffolds due to their structural similarity to aminocarboxylic acids [80]. Moreover, the stereochemistry at the phosphorus center is conserved during the reaction.

The reaction could also be performed with CH_2_Br_2_ and CH_2_I_2_ as the C1 building blocks and DMSO or MeCN as solvents. The reaction works very well with tertiary amines (both symmetrical and asymmetrical) and with some bulky primary and secondary amines (such as *n*-octyl-, dibutylamine, and *N*-methyl-*N*-butylamine). When applying primary or secondary amines are used, milder reaction conditions (75 °C) are needed.

**Scheme 28:**
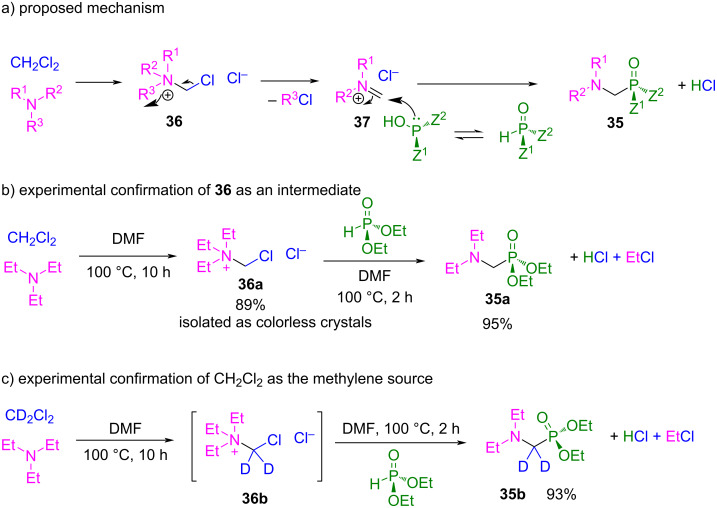
a) Proposed mechanism for the three-component reaction of dichloromethane, amine and phosphorus compound to α-amino phosphorus products **35** with conserved phosphorus stereochemistry. b) Confirmation of **36** as a reaction intermediate. c) Isotope experiment for confirmation of the role of the dihaloalkane.

The selectivity of the reaction shown in Scheme 27 could be explained according to the proposed mechanism (Scheme 28a).

The first step is the generation of the trialkyl(chloromethyl)ammonium chloride species **36** from the amine compound and CH_2_Cl_2_. Then, **36** decomposes by a cleavage of an N–C bond, where the stability of the leaving carbocation is the main factor that affects the rate of this step. Next, intermediate **37** is attacked by the phosphorus compound, giving product **35** with retention of the configuration. This mechanism was confirmed when compound **36a** was isolated as colorless crystals from the reaction between Et_3_N and CH_2_Cl_2_ at 100 °C in DMF. Subsequently, the corresponding α-aminophosphonate **35a** was obtained by heating **36a** with an equal amount of diethyl phosphite (Scheme 28b). Furthermore, when the reaction was carried out with CD_2_Cl_2_, the corresponding deuterated product **35b** was obtained, confirming that the dihaloalkane compound is the source of the methylene unit (Scheme 28c).

Depending on the stability of the leaving carbocation, the selectivity of the R–N cleavage follows the decreasing order for the R groups: H, *t*-Bu, allyl, benzyl > methyl > primary, secondary alkyl group. Therefore, for primary and secondary amines, the N–H-bond cleavage takes place predominantly instead of the other two N–R cleavage possibilities. However, in the case of tertiary amines, the N–R cleavage depends on the stability of the carbocation generated. For example, when using an amine with two methyl groups and a benzyl or allyl group, the cleavage of the N–CH_2_Ph and N–allyl bond takes place more selective (by 85% and 67%, respectively), instead of the cleavage of an N–Me bond. This explains the high selectivity observed for some examples in Scheme 27.

Finally, this methodology is a very interesting alternative for the synthesis of P-chiral α-aminophosphorous compounds without formaldehyde due to the straightforward procedure, the good yields observed, and the absence of byproducts compared to more conventional methods (Pudovik reaction or Kabachnik–Fields MCR reaction) [72–73].

### Hexamethylenetetramine

In Ugi-type isocyanide-mediated reactions, the formation of an imine from the amine and the carbonyl component is a crucial step that, and when hampered, strongly affects the outcome of the entire process. In general, the imine readily forms when aliphatic or aromatic amines and carbonyl components are used. However, when ammonia and formaldehyde are employed, side reactions are favored leading to low yields or even to no Ugi product at all [81–82].

**Scheme 29:**
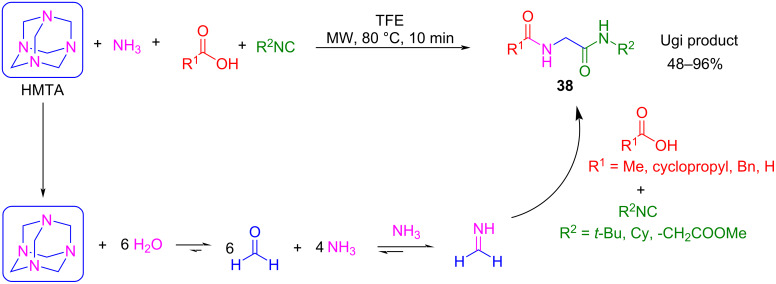
Ugi-ammonia strategy using HMTA as a formaldehyde surrogate.

Rosalba et al. showed that HMTA (hexamethylene tetramine) can be used as a formaldehyde surrogate to generate formaldehyde and ammonia in situ by heating [83]. Under these conditions, the water present in hydrated HMTA is sufficient for this hydrothermal decomposition to occur. On the other hand, an excess of ammonia must be added to the reaction mixture to shift the equilibrium towards the imine. Using this procedure, a library of acylamino acetamide derivatives **38** was synthesized from aliphatic and aromatic isocyanides and carboxylic acids, with good yields. When ammonia is replaced by methylamine, the main product incorporates methylamine as the amine component. This result indicates that HMTA is the main source of formaldehyde but not for ammonia, so the methodology can be used with other amines, too (Scheme 29).

### Glyoxylate derivatives

**Scheme 30:**
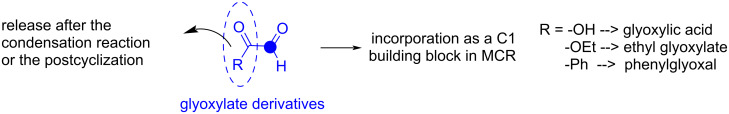
Glyoxylate and its derivatives as C1 building blocks.

Due to its low toxicity and reactivity, glyoxylic acid and its glyoxylate derivatives are among the most interesting formaldehyde surrogates in multicomponent reactions (Scheme 30). As glyoxylates are C2 structures, their use as C1 building blocks in MCRs instead of formaldehyde, requires an extra path, usually a decarboxylation process, after the condensation reaction. However, by appropriately choosing the glyoxylate derivatives (for example, ethyl glyoxylate), post-cyclization of the MCR product can be achieved, extending the universe of molecule diversity in comparison to the use of formaldehyde.

#### Glyoxylic acid as a C1 building block

Glyoxylic acid has been applied as a greener alternative to formaldehyde but also as an option to incorporate a C1 building block in multicomponent reactions where formaldehyde per se cannot react.

**Scheme 31:**
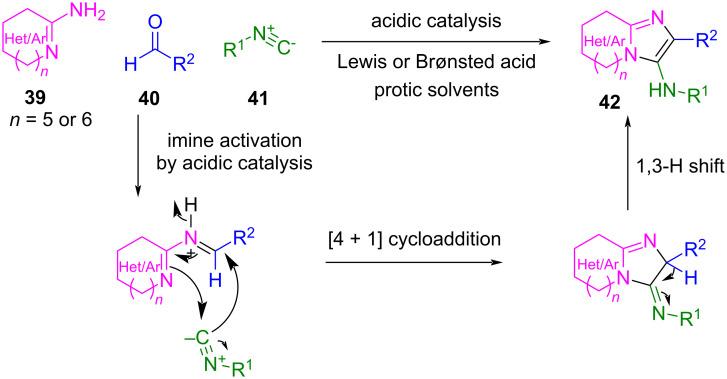
The Groebke–Blackburn–Bienaymé multicomponent reaction (GBB) and its mechanism.

For example, in the Groebke–Blackburn–Bienaymé (GBB) multicomponent reaction, a three-component reaction of heterocyclic amidines **39**, aldehydes **40** and isocyanides **41** under acidic catalysis generates heterobicyclic products **42** through a [4 + 1] cycloaddition step that ends with aromatization through a 1,3-H shift (Scheme 31) [84–85]. These compounds are highly relevant biological scaffolds for drug discovery [84].

In general, this reaction works very well with a wide variety of Lewis acids (as Sc(OTf)_3_ and MgCl_2_) and Brønsted acids (e.g., NH_4_Cl and acetic acid) and with a wide variety of isocyanides, aldehydes, and amidines. However, strikingly, the use of formaldehyde as the C1 building block is not always successful. In the few cases where the reaction proceeded as expected, low yields were obtained accompanied with several byproducts that are difficult to separate [86–88].

**Scheme 32:**
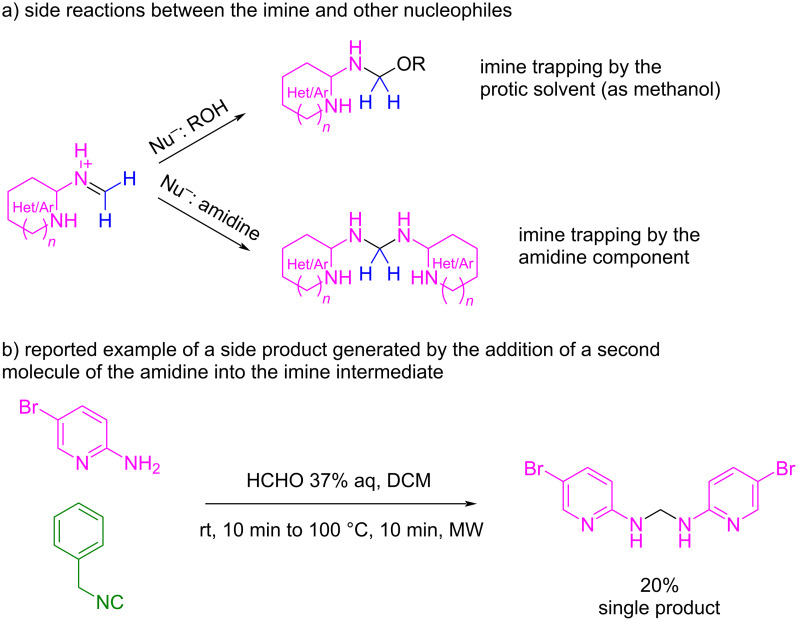
a) Byproducts in the GBB multicomponent reaction (GBB) when formaldehyde is used as the carbonyl component and b) a reported example [91].

Probably, the high reactivity of the imines generated by formaldehyde leads to their polymerization [84] or even to the incorporation of other nucleophiles present in the reaction mixture, such as the solvent or a second molecule of the amidine component (Scheme 32) [84,89–91].

**Scheme 33:**
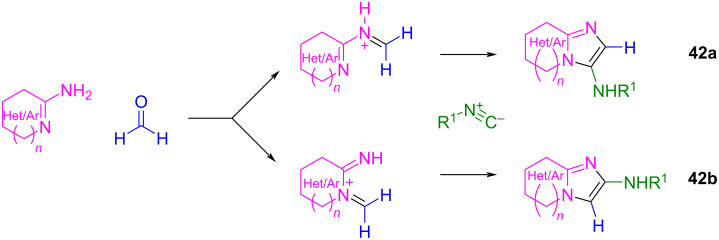
Possible regioisomers in the GBB multicomponent reaction when formaldehyde is used as the carbonyl component.

It is also known that two regioisomers can be generated during the GBB reaction depending on the nature of the amidine and the aldehyde used and, therefore, the type of imine formed [85,90]. As formaldehyde is a very reactive molecule, it will be more susceptible to form imines with either of the two nucleophilic nitrogen atoms present in the amidine component, thus leading to the production of both regioisomers **42a** and **42b** and therefore to a demanding purification process (Scheme 33).

**Scheme 34:**
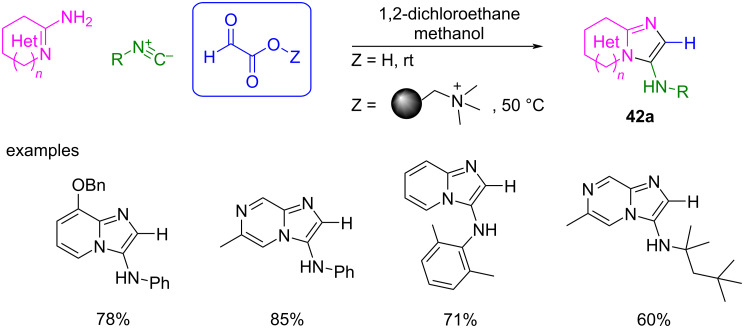
The multicomponent GBB reaction yields 2-unsubstituted 3-aminoimidazo heterocycles **42a** using MP-glyoxylate as a formaldehyde equivalent without catalyst [88].

To avoid these drawbacks, Lyon et al. optimized the reaction by using free monohydrated glyoxylic acid or MP-glyoxylate (glyoxylic acid immobilized on macroporous polystyrene resin, starting from MP-carbonate, MP-CO_3_) instead of formaldehyde (Scheme 34) [88].

When glyoxylic acid (“free” or immobilized) is used, the reaction affords 2-unsubstituted 3-aminoimidazo heterocycles **42a** as the only regioisomer with very good yields. For the immobilized glyoxylic acid, the best conditions required the use of the methanol-compatible resin, MP-carbonate. After coupling of the three components, decarboxylation at 50 °C released the product, while, when working with 'free' glyoxylic acid, decarboxylation occurred at room temperature. The mild reaction conditions for both strategies (“free” or immobilized glyoxylic acid) allows for a broad scope in terms of 2-aminoazines and isocyanide components (some examples are shown in Scheme 34).

It is important to note that these imidazo heterocycles have been reported using an alternative synthetic strategy [92], but lower efficiency in terms of yields, number of steps, and scope compared to this multicomponent methodology.

**Scheme 35:**
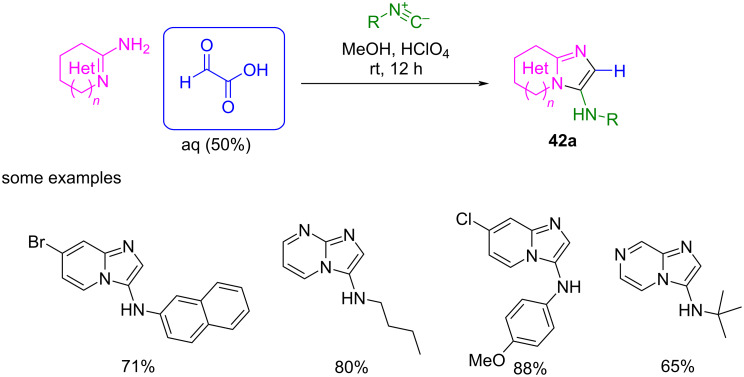
GBB multicomponent reaction to 2-unsubstituted 3-amino imidazo heterocycles **42a** using glyoxylic acid under acid catalysis.

Inspired by this previous work, Sharma et al. improved the reaction for free and immobilized glyoxylic acid, with and without acid catalysis, respectively [86,93]. They extended the scope of the reaction to a wider range of amidines and isocyanides using glyoxylic acid in 50% aqueous solution, with HClO_4_ as acid catalyst (Scheme 35) [86]. Under these conditions, the yield, the scope, and the regioselectivity of the reaction increased notably.

**Scheme 36:**
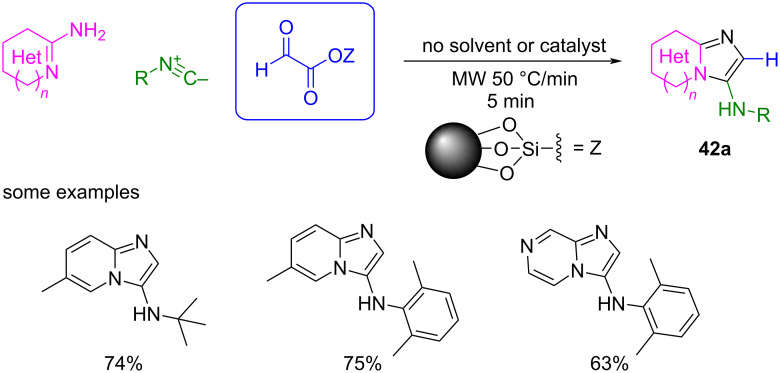
GBB reaction using glyoxylic acid immobilized on silica as formaldehyde surrogate.

Later, the same group developed an alternative method by using glyoxylic acid immobilized on silica, and the reaction conditions were optimized using microwave irradiation and avoiding the use of solvent or additional catalysts [93]. In this way, derivatives of **42a** were obtained in good yields and within shorter reaction times (Scheme 36).

**Scheme 37:**
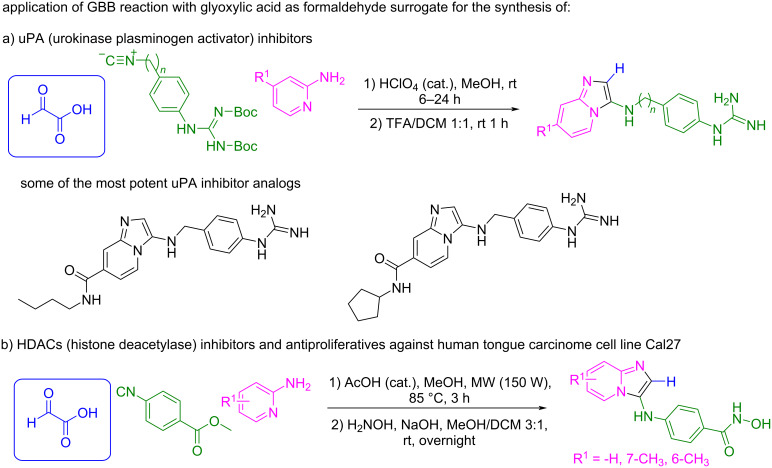
Bioactive products synthesized by the GBB reaction using glyoxylic acid.

In recent years, several groups have applied these strategies to identify products with biological activity [94–95]. Representative examples are shown in Scheme 37.

**Scheme 38:**
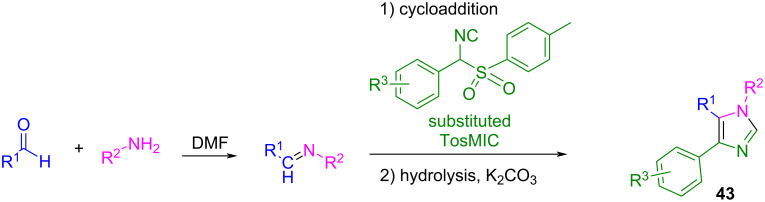
van Leusen three-component reaction to imidazoles.

On the other hand, the van Leusen three-component reaction of an aryl-substituted tosyl methyl isocyanide (TosMIC), an aldehyde, and an amine is a well-known procedure for synthesizing polysubstituted imidazoles **43** (Scheme 38). The reaction involves a cycloaddition between the isocyanide and the imine generated in situ, ending with the hydrolysis of the tosyl group.

**Scheme 39:**
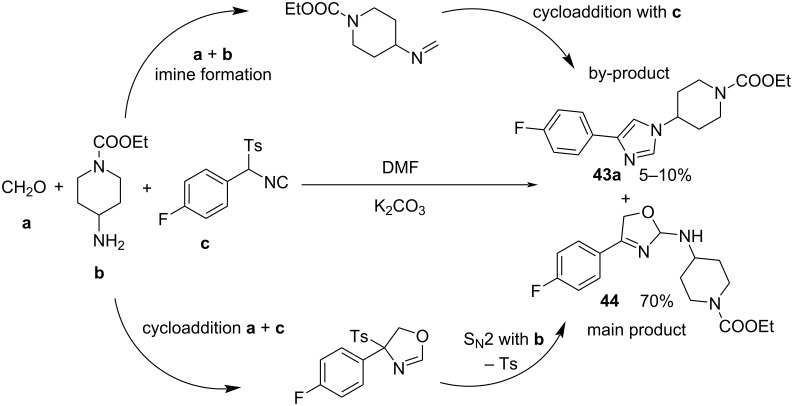
Side reaction during the synthesis of imidazoles with formaldehyde as the carbonyl compound*.*

This methodology works well for a wide variety of solvents and under mild basic conditions, since the solubility of the reagents and ease of product isolation are the factors that govern the choice. Sisko et al. used DMF/K_2_CO_3_ as the best conditions to carry out the cycloaddition of a wide variety of amines, aldehydes, and tosyl methyl isocyanides [96]. However, when the authors attempted the reaction with 37% aqueous formaldehyde, the reaction did not produce the 1,4-disustituted imidazole **43a** but instead the 2-aminooxazoline derivative **44** (Scheme 39). It was proposed that the cycloaddition between formaldehyde and the isocyanide component is preferred over the formation of the imine, probably because of the high reactivity of the carbonyl compound or the low stability of the imine. After cycloaddition, the tosyloxazoline derivative undergoes an addition at C-2 by the primary amine, followed by elimination of the toluenesulfinate moiety, producing the 2-aminooxazoline derivative **44** as the main product.

**Scheme 40:**
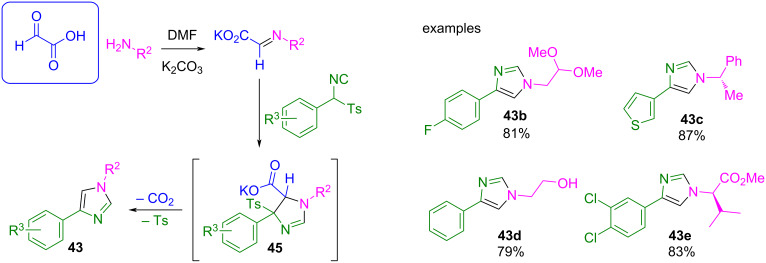
Optimization of the van Leusen three component reaction to 1,4-disubstituted imidazoles **43** using glyoxylic acid as a formaldehyde surrogate.

In this report, it was stated that this result can be avoided by replacing formaldehyde with glyoxylic acid (Scheme 40). Using similar reaction conditions, the authors obtained the desired 1,4-disubstituted imidazole derivatives **43** with very good yields and for a wide range of amines and tosyl methyl isocyanides, after a decarboxylation/elimination sequence of the putative intermediate **45**.

**Scheme 41:**
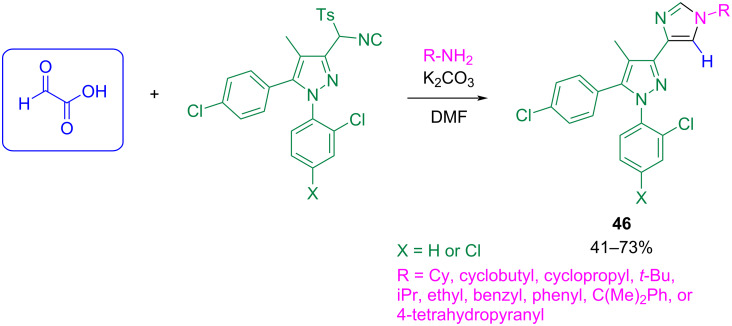
Application of the Sisko strategy [96] for the synthesis of CB1 receptor antagonist compounds [97].

This procedure was the basis for the work by Dow et al. on the synthesis of a new series of CB1 receptor antagonists (cannabinoid-1) **46** (Scheme 41) [97].

**Scheme 42:**
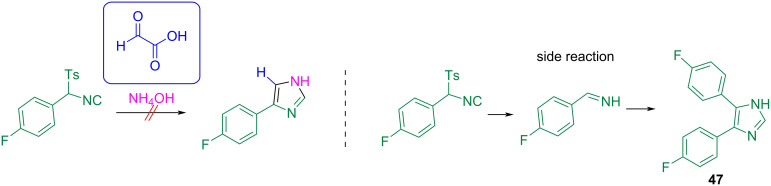
Side reaction, when NH_4_OH is used as amine component.

Unfortunately, this procedure does not work properly when amino acids or ammonia are used as amine components. In the case of ammonia, the corresponding imine was not generated, but rather a byproduct that incorporated two molecules of isocyanide **47** (Scheme 42). It was suggested that the isocyanide component decomposes to an arylimine, which undergoes a cycloaddition with another isocyanide molecule to this byproduct. However, if an amino acid is exchanged for an amino ester, the reaction affords the corresponding product **43e** (Scheme 40) as an amino acid derivative.

#### Application of glyoxylate derivatives in post-cyclization reactions as a C1 building block

**Scheme 43:**
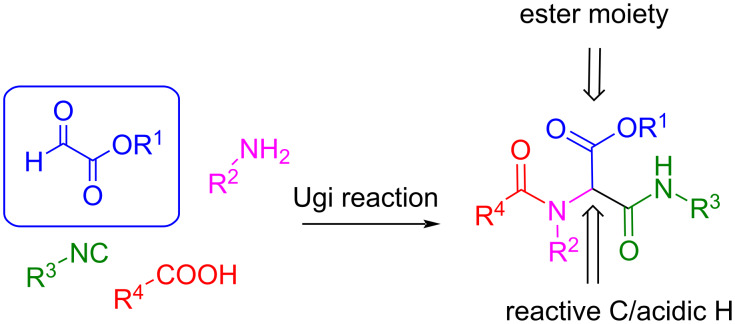
Ugi-type adducts with the ester moiety and the acidic CH to be used for post-cyclization sequences.

Glyoxylate derivatives have been used in Ugi- and Passerini-type reactions, since the adduct generated in both cases has two reactive centers for post-cyclization possibilities: the ester moiety and the α-carbon to the peptide carbonyl group. Both moieties result from the glyoxylate compound (Scheme 43).

There are many examples in which the ester moiety opens the possibility of a further intramolecular cyclization with a nucleophile (for example, a protected amine in an Ugi/deprotection/cyclization sequence [98–104]), or with a carbanion generated by a strong base in an Ugi/Dieckmann cyclization [105]. In all these cases, the glyoxylate derivative incorporates two carbon atoms into the final product, thus serving as a C2-building block.

**Scheme 44:**
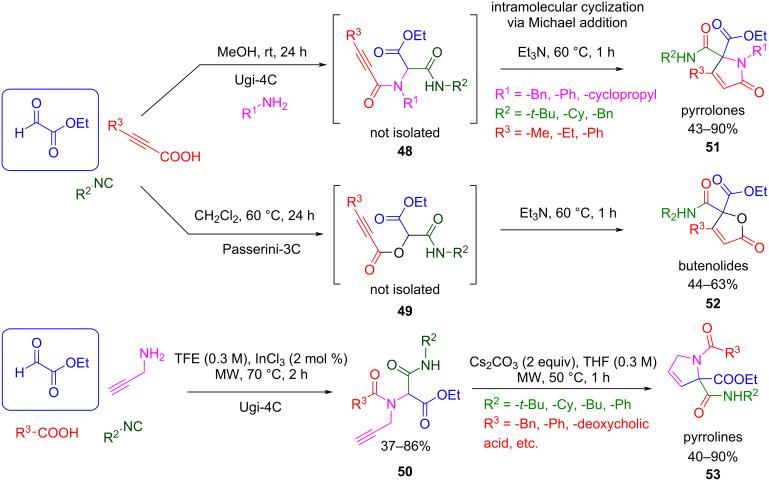
Ugi/cycloisomerization process to pyrrolones **51**, butenolides **52**, and pyrroline **53**.

Similarly, the ester group increases the acidity of the proton in the C-α position, which leads to a stable carbanion with even mild bases, that can promote intramolecular cyclization. For example, Flores-Constante et al. [106] and Nechaev et al. [107] synthesized Ugi adducts with a propargyl group that could be used as a Michael acceptor (**48**–**50**, Scheme 44). The alkyne reacts with the in situ-generated carbanion through a 5-*endo*-*dig*-cycloisomerization process to yield a nitrogen-containing five-membered heterocycle (Scheme 44). Examples in which the propargyl group is incorporated into the amine [106] or the carboxylic acid components [107] are known, leading to different cyclization products such as pyrrolines **53** and pyrrolones **51**, respectively. The procedure was also extended to Passerini adducts to afford butenolide structures **52** after the post-cyclization process [107].

In all the cases described above, the ester group of glyoxylate remains in the final products, however, it can be released after a decarboxylative or reductive reaction (β-position), allowing the use of ethyl glyoxylate as a C1 building block.

**Scheme 45:**
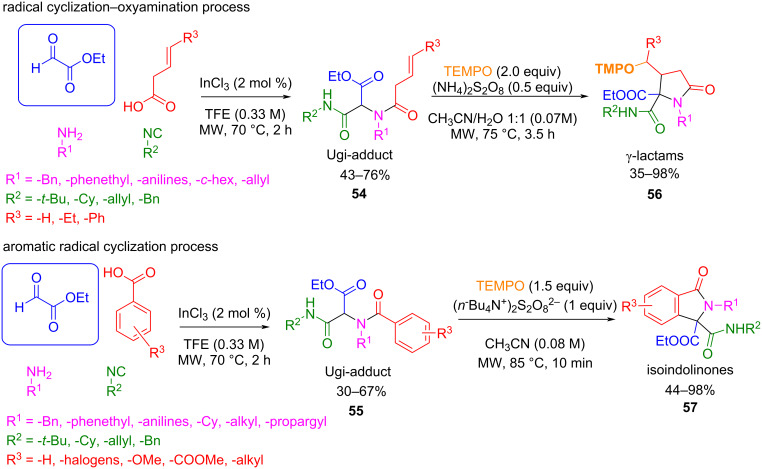
Radical cyclization reactions from Ugi adducts promoted by TEMPO.

This strategy was explored by Miranda et al., who first obtained a series of γ-lactams **56** and isoindolinones **57** using ammonium persulfate salts and TEMPO as the radical initiator/oxidant couple that promoted the intramolecular radical cyclization of suitable 1,3-dicarbonyl Ugi adducts **54** and **55** (Scheme 45) [108–109]. The stabilization of the enol in the 1,3-dicarbonyl Ugi adduct allows single-electron transfer (SET) with the anion radical species of the ammonium persulfate salt. Subsequently, the radical delocalization process gives rise to the carbon-centered radical, which follows the intramolecular cyclization onto the double bond or onto the aromatic ring depending on the case.

**Scheme 46:**
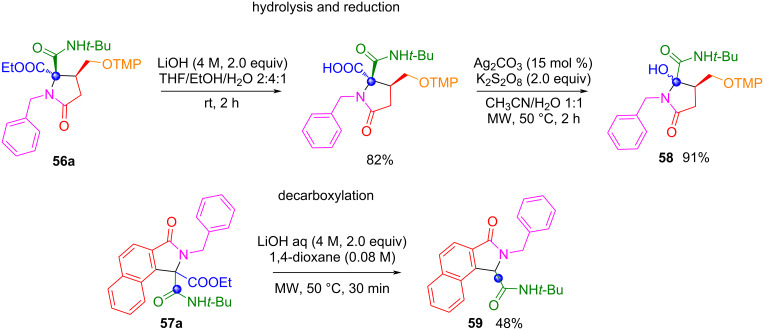
Hydrolysis and decarboxylation reactions to products with incorporation of a C1 unit of ethyl glyoxylate.

The γ-lactams **56** and isoindolinones **57** can subsequently be subjected to a decarboxylation or reductive reaction after hydrolysis of the ester group to obtain final compounds **58** and **59** whose structures incorporate only one carbon atom of the ethyl glyoxylate, and which cannot be obtained using formaldehyde (Scheme 46).

**Scheme 47:**
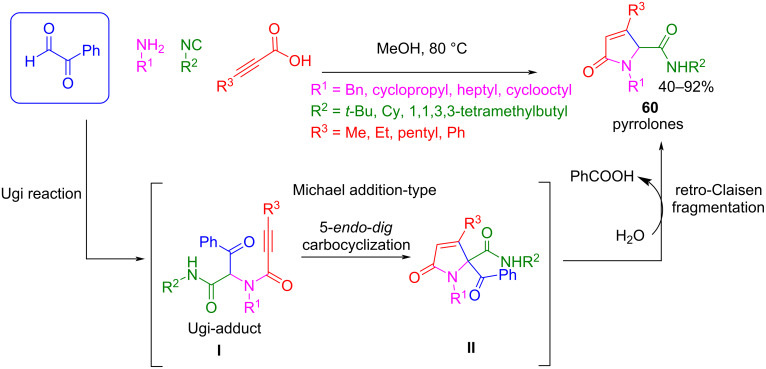
One-step synthetic route to pyrrolones **60** using phenylglyoxal.

It is important to note that, in these examples, even if the whole process is performed in one-pot, the reaction needs two steps: the generation of the Ugi (or Passerini) adduct and then the post-cyclization reaction. Interestingly, Peshkov et al. showed that when phenyl glyoxal is used instead of ethyl glyoxylate, a pyrrolone derivative **60** is obtained in one step under Ugi reaction conditions following an Ugi reaction/5-*endo*-*dig* carbocyclization/retro-Claisen fragmentation cascade reaction (Scheme 47) [110].

Although the reaction is performed under the conditions typically used in Ugi reactions (MeOH, 80 °C), in this case the outcome is determined by the presence of 3-substituted propargylic acid and phenylglyoxal as aldehyde component (Scheme 47). The Ugi adduct is not isolated, and pyrrolones **60** are spontaneously generated after cyclization of intermediate **I**, probably due to the presence of a propargyl group (conjugated with an amide) and the enolizable position favored by the presence of an additional withdrawing group (phenylcarboxy). It was proposed that, after the cyclization, intermediate **II** follows a retro-Claisen fragmentation to give the final product by releasing the phenyloxy group as benzoic acid.

It is important to note that when using paraformaldehyde instead of phenylglyoxal, only the Ugi adduct is obtained. This result suggests that the presence of the electron-withdrawing group in the carbonyl component is essential for the reaction to proceed through the cyclization step, making phenylglyoxal an excellent formaldehyde surrogate for obtaining heterocycles that could not be afforded directly.

**Scheme 48:**
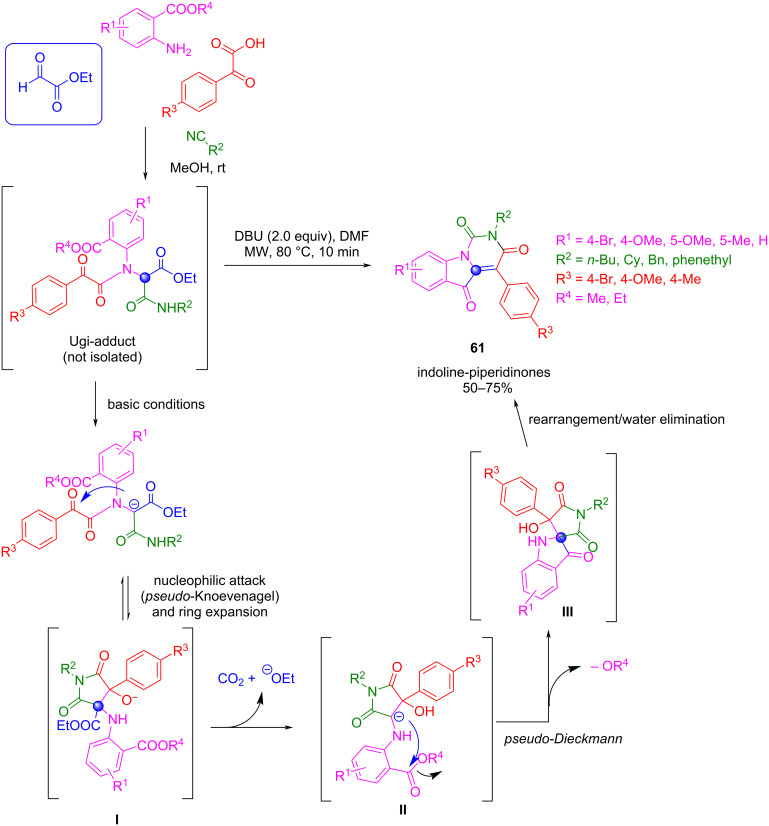
Ugi-*pseudo*-Knoevenagel-*pseudo*-Dieckmann cascade sequence for the synthesis of fused heterocycles.

In the same way, Xu’s group extended the use of stable enolate at the α-carbon in Ugi adducts as a nucleophile in *pseudo*-Knoevenagel and *pseudo*-Dieckmann cascade reactions [111–112]. Using ethyl glyoxylate, benzoyl carboxylic acids, aromatic amines having ester substituents, and a variety of aromatic and aliphatic isocyanides, these authors reported the synthesis of indoline-piperidinones fused heterocycles **61** via an Ugi/*pseudo*-Knoevenagel/ring expansion/*pseudo*-Dieckmann rearrangement cascade sequence in one pot (Scheme 48) [112].

After the completion of the Ugi reaction, the crude product was subjected without purification to basic conditions for the generation of a carbanion that attacks the carbonyl group of the ketone moiety, provoking a ring expansion that leads to intermediate **I**. Basic hydrolysis and decarboxylation at high temperature, yields a carbanionic intermediate **II** that undergoes a *pseudo*-Dieckmann reaction to give a spiro intermediate **III** which, in turn, undergoes a new rearrangement followed by water elimination to give the final product **61**.

It is important to note that, when ethyl glyoxylate was replaced by paraformaldehyde, the corresponding cyclization sequence could not proceed because the ethyl ester group is necessary to form stable carbanion **II**, which follows the intramolecular *pseudo*-Dieckmann cyclization.

**Scheme 49:**
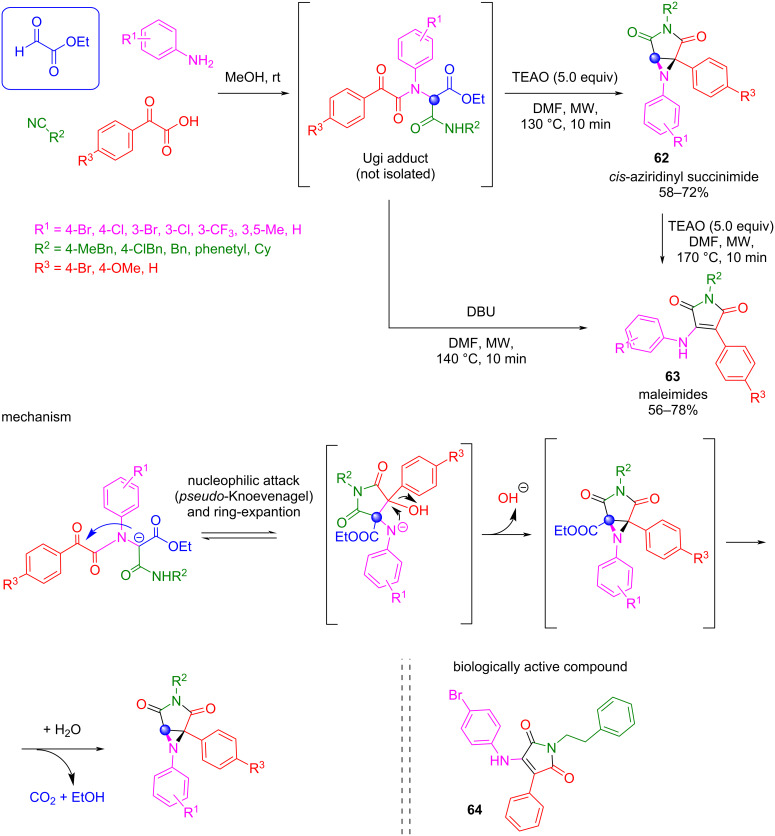
Ugi-*pseudo*-Knoevenagel reaction from ethyl glyoxylate.

In a similar context, the same authors showed that by selecting the appropriate building blocks for the synthesis of the Ugi adduct, alternative cyclization cascade reactions can be developed [111]. For example, when an aromatic amine was used lacking an additional ester group, the *pseudo*-Dieckmann intramolecular cyclization could not proceed. In this case, after the *pseudo*-Knoevenagel reaction occurred, different pathways are possible depending on the nature of the base employed (Scheme 49). When triethanolamine (TEAO) is used in DMF at 130 °C, the final product is aziridine **62**, the *cis* stereoisomer being the sole stereoisomer observed. In contrast, when treatment of the aziridinyl succinimide **62** is performed under stronger basic conditions, a ring-opening reaction is favored and maleimides **63** are obtained.

These complex one-pot cascade reactions allowed the synthesis of biologically relevant heterocyclic scaffolds. For example, one of the compounds described by these authors showed an interesting in vitro antiproliferative activity against a hepatocarcinoma cell line (**64**, Scheme 49) [111].

## Conclusion

This review summarizes the wide range of formaldehyde surrogates that can be used in MCR as a C1 building blocks to afford better reaction conditions in the synthesis of a variety of products, including imidazo compounds, pyrrolones, pyrrolines, indoline pyperidonones, propargylamines, and α-aminophosphorus compounds, among others. These products of biological relevance can be synthesized using traditional MCR reactions as GBB, Povarov, Mannich, Pudovic, Kabachnik–Fields, and isocyanide-based MCRs such as the Ugi reactions. Along with dihalomethanes, alcohols, imines, and dimethyl sulfoxide, glyoxylate and its derivatives are the most versatile formaldehyde surrogates, offering better yields, mild reaction conditions, and the potential for post-condensation reactions that are not possible with formaldehyde. In this context, Knoevenagel, Dieckmann, cyclo-isomerization, radical cyclization, and even hydrolysis and decarboxylation reactions can be implemented as post-condensation steps when glyoxylate is used in Ugi reactions. This expands the universe of possibilities to synthesize structurally more complex products via MCR.

However, despite the plethora of MCR applications wherein formaldehyde has been replaced with alternative C1 building blocks, there remain unexplored MCR reactions where this approach was not studied. This review will open new avenues for the MCR community, both in terms of applying novel formaldehyde surrogates and expanding the range of MCR reactions amenable to these substitutes.

## Data Availability

Data sharing is not applicable as no new data was generated or analyzed in this study.
